# Longitudinal changes in implant stability assessed by resonance frequency analysis and marginal bone remodeling around immediate implants: A retrospective cohort study

**DOI:** 10.34172/japid.4325

**Published:** 2026-09-12

**Authors:** Giovanni Battista Menchini-Fabris, Stefano Trasarti, Saverio Cosola, Tommaso Grandi, Soheil Bechara, Paolo Toti

**Affiliations:** ^1^Department of Psychology and Health Sciences, Pegaso University, Naples, Italy; ^2^San Rossore Dental Unit, Pisa, Italy; ^3^Tuscan Dental Institute, Versilia Hospital, Lido di Camaiore, Italy; ^4^School of Dentistry, Saint Camillus International University of Health and Medical Sciences, Rome, Italy; ^5^Private Practice, Modena, Italy; ^6^Private Practice, Vilnius, Lithuania

**Keywords:** Dental implants, Immediate dental implant loading, Osseointegration, Prosthodontics, Resonance frequency analysis

## Abstract

**Introduction:**

Resonance frequency analysis (RFA) provides a non-invasive and reproducible method for quantifying implant stability using the implant stability quotient (ISQ). Evidence on the influence of prosthetic configuration and anatomical location on ISQ changes during healing remains limited. This retrospective cohort study evaluated longitudinal changes in implant stability and investigated the influence of different implant collar configurations.

**Methods:**

Sixty-five patients (431 implants) treated with immediate implant placement and fixed prosthetic rehabilitation were included. Implant stability was assessed using ISQ values at placement and before definitive restoration. Implants were classified into four collar design groups, including an anodized collar configuration. Patient-level transition analyses were conducted using ISQ stability classes (red<60, yellow 60‒69, green≥70), and implant-level mixed-effects regression models, with patient included as a random effect, accounted for clustering within patients. Secondary outcomes included marginal bone level change, implant survival, prosthetic success, and peri-implant soft tissue indices. Significance was set at *P*<0.05.

**Results:**

ISQ values increased during healing by approximately six units. Patients rehabilitated with splinted restorations showed a higher rate of improvement in stability class than those receiving non-splinted restorations (46.7% vs. 20.0%; *P*=0.041); however, prosthetic configuration was not independently associated with baseline or postoperative ISQ values in adjusted mixed-effects analyses. Mixed-effects models identified posterior maxillary location as a significant negative predictor of ISQ, whereas implant diameter was positively associated with stability. Collar design showed no significant independent association with ISQ outcomes.

**Conclusion:**

Longitudinal RFA monitoring detected meaningful changes in implant stability during healing. Although splinted restorations showed more frequent improvements in stability class in unadjusted analyses, prosthetic configuration was not independently associated with ISQ values after multivariable adjustment. Therefore, the present findings do not support a definitive effect of splinting on implant stability. Anatomical location and implant diameter appeared to be the main determinants of ISQ values, whereas prosthetic configuration and collar design showed limited influence on implant stability outcomes.

## Graphical Abstract

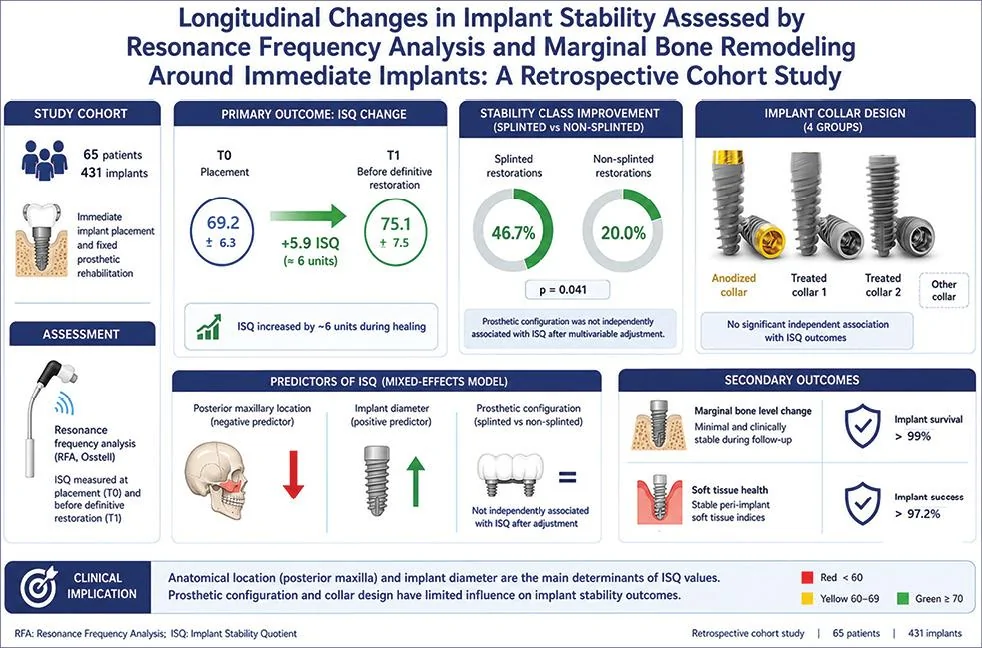

## Introduction

 Dental implant therapy is a highly predictable treatment for the rehabilitation of partially and completely edentulous patients. Advances in implant surfaces, biomaterials, and surgical protocols have improved the long-term success of implant-supported prosthetic treatments and expanded treatment options for patients with complex hard and soft tissue deficiencies.^1^

 A key determinant of successful implant therapy is the achievement and maintenance of adequate implant stability during the healing phase. Although implant stability is closely related to osseointegration, the two concepts are not synonymous. Implant stability reflects the biomechanical and biological interaction at the bone‒implant interface and represents a prerequisite for predictable osseointegration.^2,3^ From a biological and clinical standpoint, implant stability is commonly divided into two distinct components: primary stability, which derives from the mechanical engagement of the implant within the surrounding bone at the time of insertion, and secondary stability, which gradually develops as a result of bone remodeling and biological integration at the implant surface.^2,3^

 The transition from primary to secondary stability represents an important phase of osseointegration. During healing, mechanical stability may temporarily decrease because of bone remodeling before increasing again as biological anchorage develops. Monitoring implant stability during this period may support loading decisions and reduce the risk of early implant failure.

 Several methods have been proposed to evaluate implant stability, including insertion torque, removal torque, mobility assessment, percussion tests, and peri-implant radiographic evaluation.^4,5^ However, many are operator-dependent, invasive, or poorly reproducible during longitudinal follow-up, highlighting the need for objective and repeatable monitoring tools.

 Among the diagnostic techniques introduced in recent decades, resonance frequency analysis (RFA) has gained widespread acceptance as a reliable method for evaluating implant stability.^6^ This technique assesses the stiffness of the implant‒bone complex by measuring the resonance frequency generated when a transducer attached to the implant is stimulated. The resulting resonance frequency is automatically converted to the implant stability quotient (ISQ), a numerical scale ranging from 0 to 100, where higher values indicate greater implant stability.^7^

 RFA is a non-invasive, repeatable technique that provides a quantitative, operator-independent measure of implant stability throughout the healing process without interfering with biological processes. A major advantage of RFA is its ability to monitor implant stability longitudinally, providing information on biomechanical changes associated with bone remodeling during healing. Although these changes may reflect ongoing biological healing, RFA provides an indirect assessment of implant stability.^8‒10^

 However, ISQ values may be influenced by numerous factors related to implant characteristics and local anatomical conditions. Variables such as implant length, diameter, macro-design, surface properties, and surgical technique may affect the mechanical engagement between the implant and the surrounding bone.^8,9,11^ In addition, patient-level characteristics and site-specific anatomical factors, including the maxillary or mandibular location and anterior-posterior position, may substantially influence implant stability measurements.^8,9,11^

 In addition to implant macrogeometry and anatomical factors, increasing attention has been directed toward the influence of implant surface characteristics on peri-implant healing. Surface modification techniques, including sandblasting, acid etching, plasma spraying, and anodic oxidation, have been developed to optimize the biological interaction between titanium implants and surrounding tissues.^12^ Among these approaches, anodization modifies the titanium oxide layer and has been associated with enhanced protein adsorption, improved cell adhesion, accelerated osseointegration, and favorable soft-tissue integration.^13-15^

 A further development of this concept is represented by implant systems characterized by different collar surface configurations. While the implant body is primarily designed to maximize bone integration, the collar region directly interacts with peri-implant soft tissues and crestal bone, potentially influencing the establishment of the biological seal and the long-term maintenance of peri-implant health.^16^ Although anodized implants have demonstrated high survival rates and favorable clinical outcomes in medium- and long-term studies, limited information is available regarding the behavior of different collar surface configurations during the healing phase and their potential relationship with implant stability dynamics measured by RFA.^17,18^

 Longitudinal ISQ monitoring is generally more informative than isolated measurements because it allows assessment of implant stability during healing and may help identify implants with delayed integration or atypical healing patterns.^10^ Longitudinal ISQ trends may provide additional information beyond single measurements regarding implant stability during healing.

 Implant stability assessment is particularly relevant in immediate implant placement, which offers reduced treatment time and preservation of hard and soft tissues but may present greater variability because implants are inserted into fresh extraction sockets.^19^

 The timing of prosthetic loading is a key factor in implant therapy. While immediate or early loading may provide functional and aesthetic benefits, inadequate implant stability can increase the risk of failure.^20^ Higher failure rates have been reported for early loaded implants, especially in cases of poor bone quality or reduced primary stability.^20^ In addition, premature loading may negatively affect osseointegration through biomechanical stress and inflammatory responses.^21^

 In this context, longitudinal ISQ monitoring may support clinical decision-making by helping clinicians identify implants that may require prolonged healing or closer monitoring before prosthetic loading.

 Implant stability may also be influenced by the prosthetic configuration. Splinted restorations mechanically connect multiple implants and may alter load distribution compared with single-implant restorations.

 This retrospective cohort study investigated longitudinal changes in implant stability in patients undergoing immediate implant placement and fixed prosthetic rehabilitation using ISQ measurements recorded at placement and follow-up. The influence of prosthetic configuration, anatomical location, and different implant collar surface designs on implant stability and peri-implant tissue outcomes was also evaluated.

 The primary objective was to evaluate longitudinal changes in implant stability using continuous ISQ measurements recorded during healing. Transitions between predefined ISQ stability categories were analyzed only as secondary descriptive outcomes to facilitate clinical interpretation. Secondary analyses investigated whether prosthetic configuration, anatomical location, and implant collar surface design were associated with differences in implant stability and peri-implant clinical outcomes.

## Methods

###  Study Design and Sample Description

 This study adhered to the STROBE reporting guidelines. A retrospective cohort of consecutive patients treated for prosthodontic rehabilitation between January 2020 and December 2023 in an affiliated clinic operating within the National Health Service (San Rossore Dental Unit, Pisa, Italy) was analyzed. Only patients who completed prosthetic treatment and for whom complete clinical and radiographic records were available were included. Consequently, only implants with both baseline and follow-up ISQ measurements were included, and no imputation of missing ISQ values was necessary.

 The study population represented a convenience sample of routine clinical practice. Because treatment allocation was not randomized and prosthetic configuration was determined according to clinical indications, residual confounding cannot be excluded.

 The study design did not allow for strict standardization of implant insertion depth, which was determined intraoperatively based on anatomical conditions and clinical judgment. Therefore, longitudinal changes were considered more informative than absolute baseline values.

####  Inclusion Criteria

 Patients were included when all of the following criteria were fulfilled:

- aged ≥ 18 years
- written informed consent for data processing
- immediate implant placement in fresh extraction sockets
- fixed prosthetic rehabilitation
- availability of implant stability measurements recorded at implant placement and during follow-up
- availability of baseline and follow-up radiographs suitable for marginal bone level assessment

####  Exclusion Criteria

 Patients were excluded in the presence of:

- chronic systemic diseases known to potentially affect bone healing or osseointegration (e.g., uncontrolled diabetes, metabolic bone disorders, or immunocompromising conditions)
- augmentation procedures involving bone substitute materials
- heavy smoking (> 10 cigarettes/day)
- alcohol or drug abuse
- oral parafunctional habits
- incomplete clinical records or unreadable radiographs

All interventions were performed by experienced surgeons and prosthodontists.

 The study was conducted in accordance with the Declaration of Helsinki (1975, revised 2000) and its subsequent amendments governing biomedical research involving human subjects. All participants provided written informed consent. Ethical approval was obtained from the Regional Ethical Review Board of Saint Camillus International University of Health and Medical Sciences (Reference ID: E00930-2023).

###  Surgical and Prosthetic Procedures

 Following atraumatic tooth extraction whenever possible, titanium implants were placed in accordance with the manufacturer’s recommendations.

 Dental implants from the same brand, with several configurations (Icon/Icon Plus, Icon T, Pterigo, and Nasal; JDental Care s.r.l., Modena, Italy), were included. All implant groups belonged to the same implant system, minimizing manufacturer-related heterogeneity.

 Group A: Treated collar type 1: implants with a rough treated collar obtained through sandblasting and large-grit acid etching (SLA) and sharing the same implant body surface characteristics, macrodesign, and internal connection as the other study implants

 Group B: Treated collar type 2: implants with a non-anodized rough collar and SLA-treated implant body, sharing the same implant body surface characteristics and internal connection as the other study implants

 Group C: Anodized collar: implants with an SLA-treated implant body and a 1.5-mm anodized collar surface

 Group D: other implant types: pterygoid and nasal implants

 Groups A and C primarily differed in collar surface configuration

 Whenever clinically feasible, implants were positioned with the implant shoulder aligned with the crestal bone level.

 Provisional restorations were delivered when indicated. After 2‒3 months, definitive screw- or cement-retained porcelain-fused-to-metal or zirconium restorations were categorized as:

- non-splinted restorations, consisting of single implant-supported crowns
- splinted restorations, consisting of fixed prostheses supported by two or more implants, including partial fixed prostheses and full-arch rehabilitations

###  Variables

####  Implant Stability Assessment

 Implant stability was assessed using resonance frequency analysis (RFA), a non-invasive technique that expresses implant stability as an implant stability quotient (ISQ) ranging from 0 to 100, with higher values indicating greater stability.^6,7^ ISQ values were interpreted as indirect indicators of implant stability changes (Figure 1).

**Figure 1:**
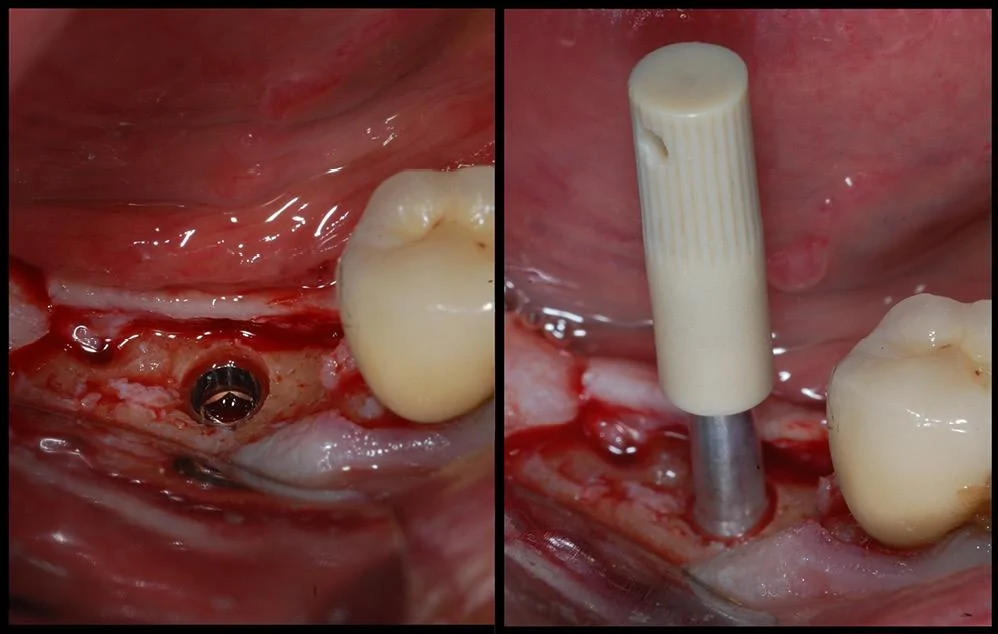
Clinical sequence of resonance frequency analysis (RFA) for implant stability measurement. Left: occlusal view of the implant at the crestal level immediately after placement, prior to measurement. Right: connection of the SmartPeg transducer to the implant for RFA assessment using the Osstell device. This non-invasive procedure allows determination of the implant stability quotient (ISQ), providing a quantitative evaluation of implant stability during the healing phase

 Measurements were obtained:

- immediately after implant placement (baseline)
- during the healing phase before definitive prosthetic delivery, which was generally performed approximately 2‒3 months after implant placement according to routine clinical protocols

Therefore, two predefined ISQ measurements were available for each implant and were used to evaluate early changes in stability. Short-term clinical outcomes were assessed separately using one-year radiographic measurements of marginal bone level.

 For splinted prostheses, the lowest implant ISQ value within the prosthetic unit was used as the representative value.

 To facilitate clinical interpretation, ISQ values were categorized into three classes:

- Green (high stability): ISQ ≥ 70
- Yellow (moderate stability): 60 ≤ ISQ < 70
- Red (low stability): ISQ < 60

The adopted thresholds corresponded to the manufacturer-recommended Osstell stability zones and have been used previously for clinical interpretation of implant stability and loading-related decision-making. Because categorization may reduce statistical information, analyses based on ISQ classes were considered complementary to the primary analyses of continuous ISQ values. Accordingly, changes were evaluated using both continuous ISQ values and ISQ categories; improvement was defined as movement to a higher category.

####  Clinical Assessment

 Peri-implant tissue conditions were evaluated from routine maintenance records.

 The following variables were collected at the 1-year follow-up:

- Gingival index (GI): The GI was used to evaluate peri-implant soft tissue inflammation on a four-point ordinal scale ranging from 0 (absence of inflammation) to 3 (severe inflammation with spontaneous bleeding).^22^
- Modified plaque index (mPI): The mPI described by Mombelli & Lang^22^ was used to evaluate plaque accumulation around implants on a four-point ordinal scale ranging from 0 (absence of plaque) to 3 (abundant plaque accumulation).^22,23^
- Modified bleeding index (mBI): The mBI described by Mombelli & Lang^22^ was assessed on four surfaces per implant-supported restoration using a four-point ordinal scale ranging from 0 (absence of bleeding) to 3 (profuse bleeding).^22‒24^

####  Radiographic Assessment

 Marginal bone levels were evaluated using standardized digital periapical radiographs obtained immediately after implant placement (T_0_) and at the 1-year follow-up (T_1_). Radiographs were acquired using an XCP-ORA positioning device (Dentsply Sirona) and a CMOS digital sensor (Schick CDR Elite, Schick Technologies). Measurements were calibrated using known implant dimensions and obtained by a blinded examiner with dedicated software (JD-igital Guide powered by RealGUIDE^TM^, JDentalCare, Modena, Italy).

 Marginal bone level (MBL) was defined as the linear distance between the implant‒abutment junction and the most coronal point of bone-to-implant contact on both mesial and distal aspects.

 Marginal bone remodeling was expressed as marginal bone level change (ΔMBL):

 ΔMBL = MBL_follow-up_ − MBL_baseline_

 As the insertion depth was not standardized, ΔMBL was considered more informative than absolute values.

###  Variables and Outcomes

####  Primary Predictor

- non-splinted restorations, consisting of single implant-supported crowns
- splinted restorations, consisting of fixed prostheses supported by two or more implants, including partial fixed prostheses and full-arch rehabilitations

####  Secondary Predictor

 The secondary predictor variable was the implant collar design:

- Group A (treated collar type 1)
- Group B (treated collar type 2)
- Group C (anodized collar)
- Group D (treated other implant types)

####  Covariates

 The following covariates were recorded: age (years), sex (male/female), arch (maxilla/mandible), tooth position (anterior/posterior), prosthetic configuration (splinted/non-splinted), implant length (mm), and implant diameter (mm).

####  Primary Outcomes

 The primary outcomes were implant stability variables: baseline ISQ, follow-up ISQ, ISQ change (ΔISQ), and transition among ISQ stability categories.

####  Secondary Outcomes

 Secondary outcomes included: marginal bone level change (ΔMBL); implant survival and success according to original and updated criteria in the absence of episodes of peri-implant mucositis or peri-implantitis; prosthetic success and failure, defined as the need for replacement of a prosthesis with an alternative prosthetic restoration; GI, mPI, and mBI with four surfaces per crown; biological complications; and mechanical complications.

 If no significant differences were detected between treated collar types 1 and 2, the groups were pooled in a secondary analysis to increase power and compare anodized versus treated collars.

####  Definitions of Failure and Success

 Implant failure was defined as the presence of at least one of the following: clinical implant mobility, radiographic peri-implant radiolucency, or implant fracture. Implant success was defined according to the original and updated implant success criteria proposed in the literature, requiring implant survival in the absence of peri-implant mucositis or peri-implantitis.^25,26^

 Prosthetic failure was defined as any prosthetic restoration requiring complete replacement with a new prosthesis.^27^

####  Complications

 Biologic complications included pain, swelling, suppuration, mucositis, and peri-implantitis. Mechanical complications included screw loosening, framework fracture, veneering material fracture, or other prosthetic technical complications.^27^

###  Statistical Analysis

 All statistical analyses were predefined and performed using MATLAB (Statistics Toolbox, MATLAB 7.0.1, The MathWorks, Natick, MA, USA). Patient- and implant-level analyses were performed to account for clustering and reviewed by a qualified biostatistician.

 For patient-level analyses, each patient was represented by a single implant selected according to predefined conservative criteria: the implant with the lowest ISQ value among all implants placed in that patient was retained. This conservative approach was adopted to reflect the weakest stability condition at the patient level. For comparative patient-level analyses, each patient was assigned to a single prosthetic configuration group; therefore, subgroup counts are not mutually exclusive. In fact, to ensure statistical independence of observations, patients rehabilitated with both splinted and non-splinted implants were assigned to the non-splinted group, which represented the smaller subgroup. For radiographic outcomes, patient-level values corresponded to median implant measurements, whereas the highest score was retained for ordinal clinical indices. Continuous implant stability outcomes were additionally analyzed at the implant level using linear mixed-effects regression models, with patients included as a random effect to account for clustering of implants within the same individual. Fixed effects included prosthetic configuration, collar configuration, arch, tooth position, implant length, implant diameter, and patient age. Baseline and postoperative ISQ values were analyzed as dependent variables. This approach allowed retention of all implant-level observations while accounting for within-patient correlation. For all mixed-effects regression models, effect sizes were expressed as regression coefficients (β) together with their 95% confidence intervals, allowing assessment of both statistical significance and clinical relevance. Consequently, statistical inference was based on mixed-effects models accounting for the hierarchical structure of the data. As only implants with complete baseline and follow-up ISQ recordings were included, the analytical dataset contained no missing values for the primary stability outcomes, and no missing-data replacement procedures were performed.

 The distribution of ISQ values was also categorized into three stability classes (red: ISQ < 60; yellow: ISQ = 60‒69; green: ISQ ≥ 70). These thresholds correspond to the manufacturer-recommended Osstell stability zones and have been adopted in previous resonance frequency analysis studies to facilitate clinical interpretation of implant stability. To evaluate whether the proportion of patients who improved in stability class differed between prosthetic configurations (non-splinted vs. splinted), a chi-squared test of independence was performed. Improvement was defined at the patient level as a transition from a lower to a higher ISQ stability category between baseline and postoperative evaluation. ISQ stability class transitions were analyzed at the patient level. These analyses provided a clinically interpretable description of changes in stability, whereas the inferential analyses were based on continuous ISQ measurements. For patient-level transition analyses, each patient contributed only a single observation, ensuring independence of observations and avoiding duplication of information from patients receiving multiple implants. To further explore the clinical relevance of changes in implant stability, the association between ΔISQ and marginal bone level change (ΔMBL) was evaluated at the patient level using Spearman’s rank correlation. Median patient-level values were used to avoid non-independence arising from multiple implants placed in the same individual.

 Normality and homoscedasticity assumptions were assessed using the Shapiro-Wilk and Brown-Forsythe tests, respectively. Continuous non-normally distributed variables were analyzed using Kruskal-Wallis tests followed by post hoc Dunn comparisons with Bonferroni adjustment. The group with other implants was included in the ISQ mixed-effects models but excluded from MBL analyses because it represented a small and clinically distinct subgroup. Categorical variables were analyzed using chi-squared tests or Fisher’s exact tests when appropriate. Survival rates and corresponding 95% confidence intervals were calculated according to the methods described by Romeo et al.^28^ and Eckert et al.^29^ Statistical significance was set at *P* < 0.05 for all analyses.

## Results

 Sixty-five patients (30 females and 35 males) receiving 431 implants were included in the study. The mean age of the cohort was 59.4 ± 13.4 years. Baseline patient and implant characteristics are summarized in Table 1, whereas a complete implant-level description is provided in Supplementary Table S1 (Supplementary file 1). Four implant groups were identified: 235 implants in group B, 107 in group C, 73 in group A, and 16 in group D. Most implants were restored with splinted prosthetic rehabilitation (390 implants), whereas 41 were restored with non-splinted restorations. Implant placement was distributed across all anatomical regions, with the posterior maxilla representing the most frequent site (166 implants), followed by the posterior mandible (133 implants), the anterior maxilla (69 implants), and the anterior mandible (63 implants) (Tables 1 and 2).

**Table 1:** Demographic and implant-related characteristics of the study population. Data are reported for the overall cohort and according to implant collar design: group A (treated collar type 1), group B (treated collar type 2), group C (anodized collar), and group D (other implant collar designs not included in groups A‒C, comprising N- and P-type implants)

**Variable**	**Overall**	**Group A**	**Group B**	**Group C**	**Group D**
Age (mean ± SD)	59.4 ± 13.4	59.2 ± 13.7	54.8 ± 13.1	64.8 ± 11.0	67.3 ± 8.7
Patients, n	65	60	30	28	9
Implants, n	431	235	107	73	16
Female/Male implants, n	179/252	83/152	45/62	42/31	9/7
Maxilla/Mandible implants, n	235/196	122/113	67/40	32/41	14/2
Anterior/Posterior implants, n	132/299	101/134	3/104	26/47	2/14
Splinted/Non-splinted implants, n	390/41	211/24	96/11	67/6	16/0
Implant diameter (mm)	3.9 ± 0.3	3.9 ± 0.3	4.2 ± 0.2	3.8 ± 0.2	3.6 ± 0.4
Implant length (mm)	12.3 ± 2.6	12.3 ± 2.3	11.4 ± 1.4	12.2 ± 2.6	18.7 ± 3.6

Values are presented as mean ± standard deviation (SD), or counts. Implant distribution according to sex, jaw (maxilla/mandible), region (anterior/posterior), splinting status, implant diameter, and implant length is also reported. The same patient could contribute implants to more than one collar-design group; therefore, the sum of patients across groups exceeds the total number of patients.

**Table 2:** Prosthetic rehabilitation data and implant stability (ISQ) outcomes. The table summarizes the number of patients and implants for each prosthetic strategy (splinted and non-splinted) and reports the distribution and median (interquartile range) for implant stability quotient (ISQ) at baseline, postoperative, and their change (ΔISQ)

**Prosthesis type**	**Patients**	**Implants**	**Baseline**	**Postoperative**
Non-splinted	20	41	72.7 (4.7)	76.1 (11.4)
Splinted	61	390	71.0 (6.6)	76.4 (7.8)
Maxillary, anterior	46	69	69.7 (3.2)	75.7 (4.7)
Maxillary, posterior	52	166	66.6 (5.4)	72.1 (5.9)
Mandibular, anterior	47	63	73.7 (1.5)	79.9 (3.1)
Mandibular, posterior	46	133	72.9 (1.9)	78.7 (4.5)

ISQ values represent patient-level summaries based on the lowest implant ISQ per patient (conservative approach). The table reports descriptive statistics stratified by prosthetic configuration and anatomical location. In this descriptive framework, patients may contribute to more than one subgroup if they received implants with different characteristics. Therefore, the number of patients within each subgroup does not necessarily correspond to the number used in comparative analyses.

###  Implant Stability Outcomes

 Descriptive stability outcomes are reported in Table 2. At the patient level, postoperative ISQ values were consistently higher than baseline values across all anatomical regions. The highest stability values were observed in mandibular sites, whereas the lowest values were recorded in the posterior maxilla.

 Changes in implant stability classes were evaluated using transition matrices based on red (ISQ < 60), yellow (60 ≤ ISQ < 70), and green (ISQ ≥ 70) categories. Overall, ISQ stability shifted toward higher categories between baseline and postoperative assessment (Table 3, Figure 2).

**Table 3:** Patient-level stability class transitions and improvement analysis (non-splinted vs. splinted)

**Stability transition (Baseline → Postoperative**	**Variable/ Comparison**	**Red**	**Yellow**	**Green**	**Total/ %**	**Test/ Statistic**
All implants	Red	8	12	0	20	Total: 65
	Yellow	0	8	13	21	
	Green	0	4	24	24	
Non-splinted	Red	0	0	0	0	Total: 20
	Yellow	0	4	4	8	
	Green	0	0	12	12	
Splinted	Red	8	12	0	20	Total: 45
	Yellow	0	4	9	13	
	Green	0	0	12	12	
Improvement rate	Non-splinted			4/20 (20.0%)		
	Splinted			21/45 (46.7%)		
Test	Chi-squared			4.16, df = 1, *P* = 0.041		

The first section reports the number of patients with implants transitioning between stability classes (red, yellow, green) from pre- to postoperative assessment. The second section shows the number and percentage of patients with at least an implant that improved for each prosthesis group (non-splinted, splinted). The third section summarizes the overall statistical test (chi-squared).

**Figure 2:**
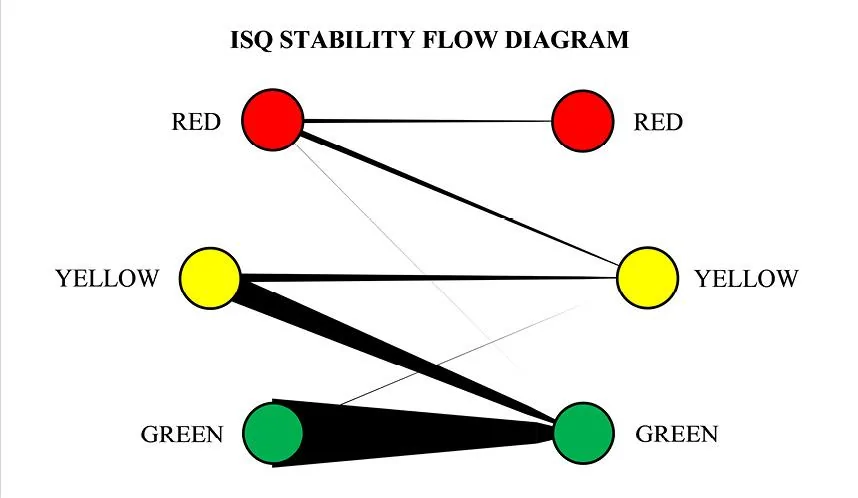
Visual representation of implant stability results. Circular transition flow diagram of ISQ stability classes (red, yellow, green) from preoperative to postoperative assessment. Node sizes reflect the relative number of patients in each class, while tapered arrows indicate transitions, with arrow width proportional to the number of patients moving between classes. Preoperative nodes are on the left and postoperative nodes on the right

 Among non-splinted restorations, 4 of 20 patients (20.0%) had at least one implant that showed an improvement in stability class, whereas 16 patients (80%) remained unchanged. In the splinted group, improvements were more frequent, with 21 of 45 patients (46.7%) demonstrating at least one implant transitioning to a higher stability class. The proportion of patients exhibiting improved implant stability was significantly higher in the splinted group than in the non-splinted group (χ^2^ = 4.16, df = 1, *P* = 0.041). However, this difference was not confirmed in the adjusted mixed-effects analyses, in which prosthetic configuration was not independently associated with ISQ values.

 Baseline and postoperative ISQs were further analyzed using linear mixed-effects regression models (Table 4; Figure 3). Implant position was the strongest predictor of stability. Compared with the reference category (non-splinted implants placed in the posterior mandible from group C), implants placed in the posterior maxilla showed significantly lower ISQ values at both baseline (β = -7.57; 95% CI: -8.71 to -6.44; *P* < 0.001) and postoperative assessment (β = -7.75; 95% CI: -9.08 to -6.42; *P* < 0.001). Likewise, implants placed in the anterior maxilla demonstrated significantly lower stability values than those placed in mandibular sites (baseline: β = -2.72; 95% CI: -4.30 to -1.14; postoperative: β = -2.98; 95% CI: -4.83 to -1.13).

**Table 4:** Linear mixed-effects regression models for baseline and postoperative Implant Stability Quotient (ISQ). Regression coefficients (β), standard errors (SE), t values, *P*-values, and 95% confidence intervals (CI) are reported

**Variables/** **Comparisons**	**β**	**SE**	**t**	***P***	**95% CI**	
ISQ baseline						
Intercept: Non-splinted - Mandibular, Posterior - Anodized (Group C)	62.043	4.246	14.613	**0.0000**	[53.722	70.365]
Splinted	0.266	0.800	0.332	0.7397	[-1.302	1.833]
Age	0.022	0.018	1.199	0.2304	[-0.014	0.057]
Length	0.171	0.115	1.484	0.1378	[-0.055	0.397]
Diameter	1.762	0.870	2.024	**0.0430**	[0.056	3.468]
Maxillary Anterior	-2.723	0.805	-3.381	**0.0007**	[-4.301	-1.144]
Maxillary Posterior	-7.574	0.579	-13.088	**0.0000**	[-8.708	-6.440]
Mandibular Anterior	0.988	0.800	1.235	0.2169	[-0.580	2.555]
Group A vs. C	0.285	0.809	0.352	0.7245	[-1.301	1.871]
Group B vs. C	-0.196	0.623	-0.315	0.7526	[-1.418	1.025]
Group D vs. C	-1.498	1.591	-0.941	0.3466	[-4.617	1.621]
ISQ postoperative						
Intercept: Non-splinted - Mandibular, Posterior - Anodized	63.879	4.980	12.827	**0.0000**	[54.118	73.640]
Splinted	-0.334	0.938	-0.356	0.7216	[-2.173	1.504]
Age	0.023	0.021	1.092	0.2748	[-0.018	0.065]
Length	0.229	0.135	1.692	0.0906	[-0.036	0.494]
Diameter	2.579	1.021	2.526	**0.0115**	[0.578	4.580]
Maxillary Anterior	-2.981	0.945	-3.156	**0.0016**	[-4.833	-1.130]
Maxillary Posterior	-7.749	0.679	-11.415	**0.0000**	[-9.079	-6.418]
Mandibular Anterior	1.011	0.938	1.077	0.2814	[-0.828	2.849]
Group A vs. C	1.477	0.949	1.556	0.1198	[-0.384	3.337]
Group B vs. C	0.084	0.731	0.115	0.9084	[-1.349	1.517]
Group D vs. C	-1.705	1.867	-0.913	0.3611	[-5.363	1.954]

Models included prosthetic configuration, implant position, age, implant length, implant diameter, and implant group as fixed effects, while patient was included as a random intercept to account for clustering of implants within the same individual. The reference category was a non-splinted implant in the posterior mandibular region from group C (anodized implants). Significant differences are indicated by *P* < 0.05.

**Figure 3:**
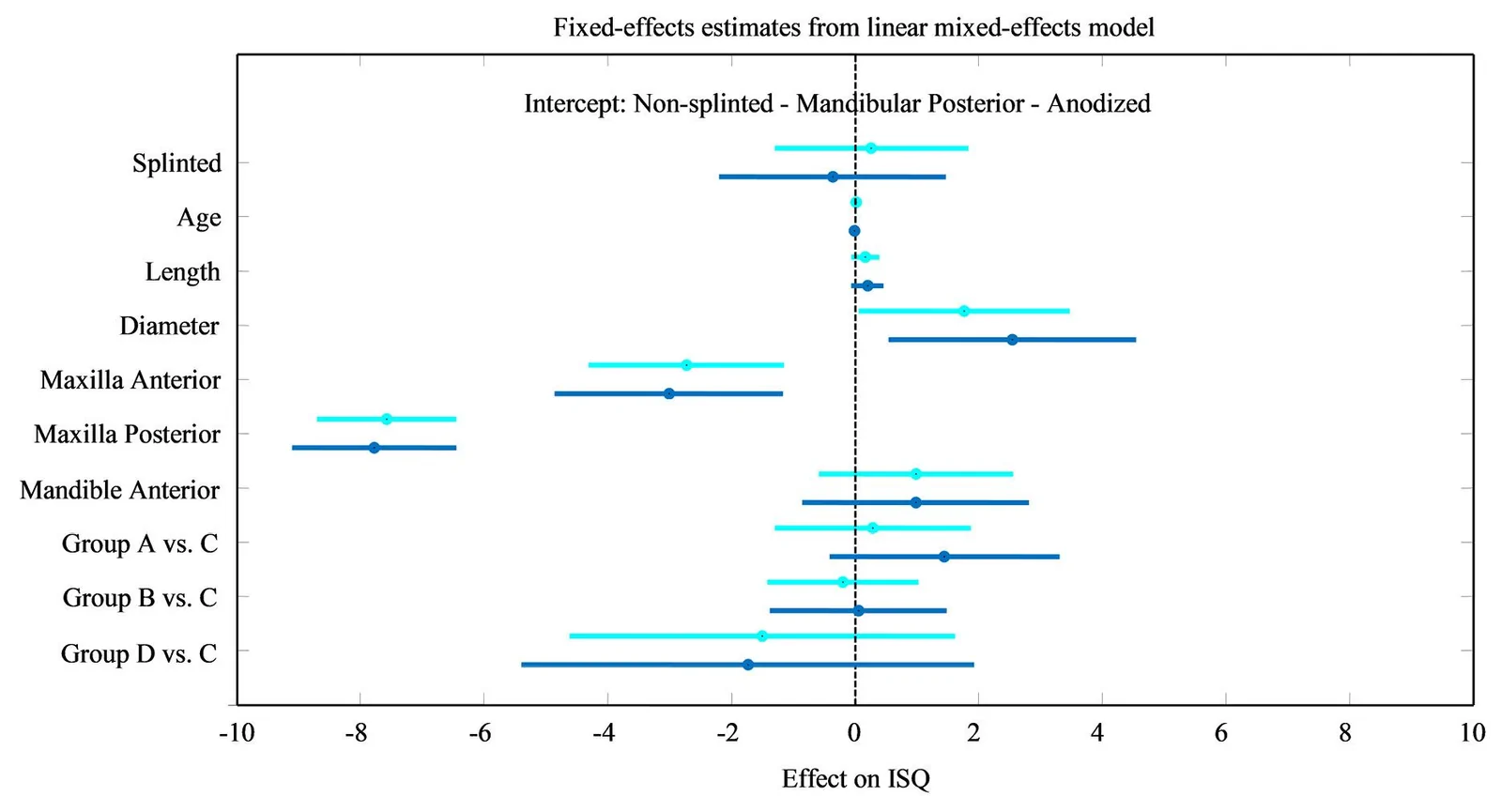
Model-predicted implant stability (ISQ) across covariates. Predicted ISQ values derived from the mixed-effects regression model are shown for each feature. Points represent the estimated marginal mean ISQ, while horizontal bars indicate the corresponding 95% confidence intervals. Cyan symbols correspond to baseline measurements, whereas blue symbols represent postoperative measurements. The intercept represents the reference condition corresponding to non-splinted implants placed in the mandibular posterior region and belonging to group C (anodized collar). Regression coefficients for the remaining anatomical regions indicate deviations from this reference category. The model also showed a significant positive association between implant diameter and ISQ values

 Implant diameter showed a significant positive association with ISQ values both at baseline (β = 1.76; 95% CI: 0.06 to 3.47; *P* = 0.043) and postoperatively (β = 2.58; 95% CI: 0.58 to 4.58; *P* = 0.012). Prosthetic configuration, patient age, implant length, and implant collar design were not significantly associated with ISQ values.

###  Marginal Bone Level Outcomes

 Radiographic evaluation showed stable marginal bone levels throughout the observation period (Figure 4). Representative panoramic radiographs obtained during follow-up are presented in Supplementary Figure 1. At baseline, most implants were positioned within the supracrestal range, between 0 and +1 mm relative to the implant‒abutment junction. Although treated collar type 2 implants showed a tendency toward a more equi-crestal positioning at placement, baseline MBL distributions were broadly comparable among collar designs.

**Figure 4:**
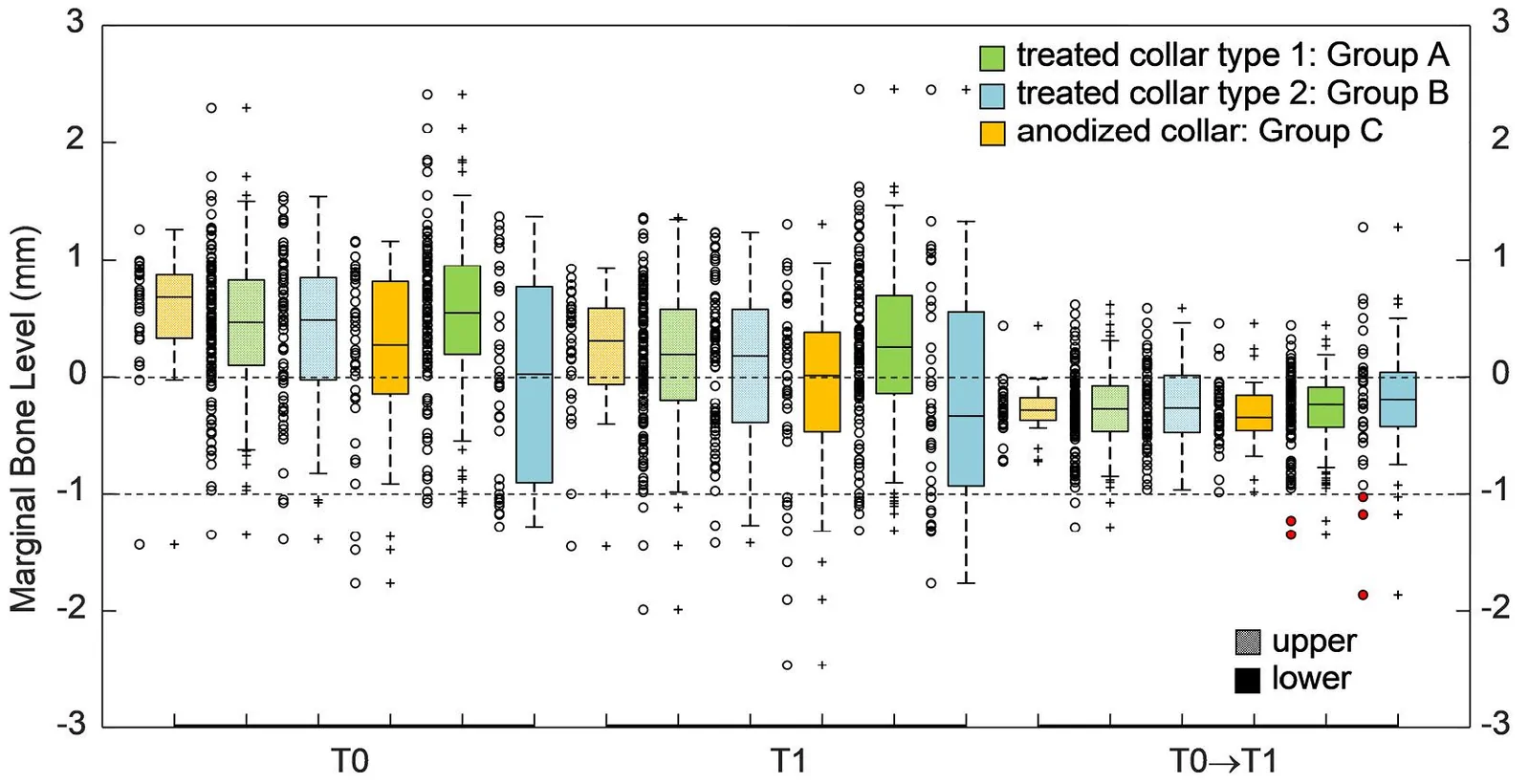
Plot showing results of radiographic evaluation of marginal bone levels (MBL, mm) around implants belonging to group A (treated collar type 1), group B (treated collar type 2), and group C (anodized collar) after implant placement (T_0_) and one year after (T_1_) for individual implants, stratified by collar type. Implants belonging to group D were not included in this analysis because they represented a small and clinically distinct subgroup consisting predominantly of long implants placed in nasal and pterygoid anatomical sites. The vertical axis represents MBL relative to the implant abutment junction; negative values indicate subcrestal bone positioning. Red circles indicate surviving implants classified as unsuccessful, defined as marginal bone loss > 1 mm at the 1-year follow-up. Data are presented using box-and-whisker plots, with external values plotted on the x-axis. The plot shows the median, interquartile range (IQR), and percentiles for each group at different time points (T_0_, T_1_, and T_0_→T_1_). The groups are categorized by the type of surface treatment: anodized (yellow), type 1 (green), and type 2 (blue)

 At the one-year follow-up, all collar groups exhibited modest reductions in MBL relative to baseline. Median changes ranged from approximately −0.18 mm to −0.29 mm, indicating limited marginal bone remodeling during function. No significant differences in ΔMBL were observed between anodized and treated collar designs, suggesting comparable bone-level behavior irrespective of collar surface characteristics. The association between median ΔISQ and median ΔMBL values was evaluated using a patient-level correlation analysis. No significant association was observed (Spearman’s *P* = 0.011, *P* = 0.929), indicating that the magnitude of ISQ increase during healing was not related to marginal bone level changes at the 1-year follow-up.

 Five implants exhibited bone loss > 1 mm but remained functional; no cases of peri-implant mucositis or peri-implantitis were recorded. Peri-implant soft tissue parameters showed generally favorable conditions across all collar groups. Detailed distributions of the GI, mPI, and mBI are reported in Supplementary Table S2 (Supplementary file 2). For the GI, score 0 was observed in 40.5% of patients in group A, 63.6% in group B, and 70.6% in group C, whereas only a small proportion of patients exhibited score 2 values (5.4%, 9.1%, and 5.9%, respectively), and no score 3 values were recorded. Similarly, mPI distributions were characterized by a predominance of low scores, with score 0 recorded in 32.4%, 54.5%, and 41.2% of patients in groups A, B, and C, respectively. No statistically significant differences were detected between collar groups for either GI (overall *P* = 0.0864) or mPI (overall *P* = 0.4018), indicating comparable peri-implant soft tissue health and plaque control across the investigated implant designs.

 The anodized collar group showed a more favorable distribution of modified mBI scores. Specifically, 82.4% of patients in group C had a bleeding score of 0, compared with 51.4% and 45.5% in groups A and B, respectively, and no score 2 values were recorded in the anodized collar group. Although the overall comparison between the three groups was not statistically significant (*P* = 0.0629), pooling the two collar groups in a secondary exploratory analysis showed significantly lower bleeding scores for anodized collars (82.4% vs. 50.0% with a score of 0; Fisher’s exact test, *P* = 0.0239).

###  Implant Success and Survival

 Implant success and survival outcomes are summarized in Table 5. Three early implant failures were recorded, all involving short (6 mm) implants placed in the posterior mandible of a single patient. Two failures occurred in group A and one in group C. The failed implants were subsequently replaced and excluded from longitudinal analyses.

**Table 5:** Implant success and survival rates: event distribution table among groups

**Group A (treated collar type 1)**								
Yrs	Loaded	Early failure	At risk	Success	Survival event	Failure event	Success rate (%)	Survival rate (%)
0‒2	235	2	233	229	233	0	98.3 (95% CI: 96.6‒99.9)	100
**Group B (treated collar type 2)**								
Yrs	Loaded	Early failure	At risk	Success	Survival event	Failure event	Success rate (%)	Survival rate (%)
0‒2	107	0	107	104	107	0	97.2 (95% CI: 94.1‒100)	100
**Group C (anodized collar)**								
Yrs	Loaded	Early failure	At risk	Success	Survival event	Failure event	Success rate (%)	Survival rate (%)
0‒2	73	1	72	72	72	0	100	100
**Group D (N- and P-type long implants in nasal and pterygoid sites)**								
Yrs	Loaded	Early failure	At risk	Success	Survival event	Failure event	Success rate (%)	Survival rate (%)
0‒2	16	0	16	16	16	0	100	100

 Success rates ranged from 97.2% to 100% across implant collar groups. Group A demonstrated a cumulative success rate of 98.3% (95% CI: 96.6‒99.9), whereas groups B and C achieved success rates of 97.2% and 100%, respectively. Group D also showed a success rate of 100%. After excluding early failures, implant survival reached 100% across all groups during the follow-up period.

## Discussion

 This retrospective cohort study showed that implant stability increased during healing, marginal bone remodeling remained limited regardless of collar design, and implant survival was excellent across all groups. ISQ values increased between placement and follow-up across all anatomical regions, supporting the expected transition from primary mechanical stability to secondary biological stability during osseointegration. The greatest increase occurred in the posterior maxilla, suggesting that implants placed in less favorable bone conditions can achieve substantial biological stabilization during healing. These findings are consistent with previous studies showing increases in ISQ values after the early remodeling phase.^30,31^

 Patients rehabilitated with splinted prostheses more frequently showed improvements in ISQ stability class than those receiving non-splinted restorations (46.7% vs. 20.0%); however, this association was not confirmed after multivariate adjustment. The observed categorical differences should be regarded as descriptive findings. Although the patient-level analysis identified a significant association, the multivariable analyses did not support prosthetic configuration as an independent predictor of implant stability. Consequently, the present findings do not provide robust evidence that splinting directly improves implant stability. The apparent discrepancy between the patient-level categorical analysis and the implant-level adjusted models likely reflects differences in outcome definition and statistical adjustment. Because the mixed-effects models accounted for clustering within patients and adjusted for relevant covariates, greater weight should be attributed to these analyses when interpreting the potential influence of prosthetic configuration on implant stability. Anatomical location appears to be the strongest determinant of implant stability, with implants placed in the posterior maxilla showing approximately 7.5 ISQ units, which were lower than implants placed in the posterior mandible (95% CI: approximately 6‒9 ISQ units lower) at both baseline and postoperative assessments. As the influence of anatomical location and bone characteristics on resonance frequency measurements has been consistently reported in previous studies, this observation appears to be a confirmatory finding.^9,32^ In the present investigation, these associations remained significant after multivariable adjustment.

 Marginal bone remodeling remained limited throughout follow-up (median ΔMBL: −0.18 to −0.29 mm), with no significant differences between collar designs. These findings are consistent with previous reports indicating that modern implant surfaces provide stable peri-implant bone conditions irrespective of collar surface treatment.^13‒15,18^

 Peri-implant soft tissue conditions remained favorable across all groups. Although anodized collars showed lower bleeding scores in an exploratory pooled analysis, this finding should be interpreted with caution given the limited sample size. Implant survival remained 100% after excluding three early failures, and success rates exceeded 97% across all collar groups, consistent with previous reports.^18,33,34^ Longitudinal RFA monitoring proved useful for documenting changes in stability during healing; however, ISQ values should be interpreted alongside clinical and radiographic findings, as they are surrogate biomechanical markers rather than direct measures of peri-implant bone preservation or long-term treatment success.

 The increase in ISQ values observed in the present study is consistent with the expected transition from primary mechanical stability to secondary biological stability during healing. Since ISQ measurements were obtained at implant placement and again before definitive restoration approximately 2‒3 months later, the present findings primarily describe the early phase of osseointegration rather than long-term stability changes after functional loading. However, peri-implant bone behavior was additionally evaluated through a radiographic assessment of marginal bone level at the one-year follow-up, providing a short-term clinical perspective on implant performance. Resonance frequency analysis provides a non-invasive and repeatable method for monitoring these changes over time and has been widely adopted for longitudinal assessment of implant stability. The progressive increase in ISQ values observed in the present cohort agrees with previous reports describing similar temporal patterns during osseointegration.^30,31^

 Anatomical location was the strongest determinant of implant stability, with significantly lower ISQ values in the maxilla, particularly in posterior sites. Previous studies have demonstrated the influence of bone density and bone morphology on resonance frequency measurements. Salimov et al.^32^ reported a significant correlation between higher bone density and increased implant stability, whereas Sim and Lang^9^ demonstrated that implants placed in denser bone structures generally show higher ISQ values during healing. These findings support the interpretation that differences observed between maxillary and mandibular sites primarily reflect variations in bone quality and microarchitecture. Anatomical location may also act as a surrogate for other clinically relevant factors, including prosthetic treatment planning, extraction socket characteristics, and implant selection strategies. The observed anatomical associations should not be interpreted as purely causal effects of site location alone.

 Implant diameter was also positively associated with ISQ values at both baseline and follow-up. The magnitude of this effect was approximately 1.8 ISQ units at baseline and 2.6 ISQ units at follow-up for each additional millimeter of implant diameter. Larger implant diameters may increase the implant‒bone contact area and improve mechanical engagement with the surrounding bone, thereby contributing to greater stability. This finding is consistent with previous investigations evaluating determinants of implant stability.^11^

 Previous studies have reported similar observations, indicating that although splinted restorations may alter load distribution among implants, their direct influence on implant stability appears limited when other biological and anatomical determinants are considered.^33^ Implant stability during healing seems to be primarily driven by site-specific bone characteristics and implant-related factors rather than prosthetic configuration alone.

 The present study has several limitations. Its retrospective design and relatively limited sample size of the non-splinted group may restrict the generalizability of the results. Because prosthetic configuration was determined according to clinical judgment rather than random allocation, treatment-selection bias and residual confounding cannot be completely excluded. In particular, the decision to provide splinted or non-splinted restorations may have been influenced by anatomical location, perceived bone quality, case complexity, prosthetic requirements, and clinician preference. Although multivariable mixed-effects models adjusted for principal measured covariates, unmeasured factors such as bone density, insertion torque, extraction socket morphology, and clinician preference may still have influenced the observed outcomes. In particular, anatomical location may partially reflect underlying differences in bone quality and treatment planning variables that were not directly measured in the present study.

 Since prosthetic configuration was determined according to clinical requirements rather than random allocation, the observed differences between splinted and non-splinted restorations should not be interpreted as evidence of a direct treatment effect. Residual treatment selection bias and unmeasured confounding remain possible despite statistical adjustment.

 Another limitation concerns the duration and structure of implant stability follow-up. Only two ISQ measurements were available for each implant (baseline and pre-restorative assessment at approximately 2‒3 months), which precluded evaluation of more complex stability trajectories during healing. The study may not capture the complete long-term evolution of implant stability beyond the early phases. Implant stability is known to change over time as bone remodeling continues after functional loading, and longer observation periods may provide additional insights into the long-term behavior of ISQ values.

 However, short-term clinical behavior was additionally assessed through radiographic marginal bone level evaluation at the one-year follow-up. The observed ΔMBL values were limited and remained within ranges commonly reported in the contemporary implant literature, supporting the overall clinical stability of the investigated implants despite the absence of extended longitudinal ISQ monitoring. Furthermore, resonance frequency analysis provides a useful longitudinal assessment of implant stability during healing. However, ISQ values should be interpreted as surrogate biomechanical markers. Similarly, improvements in ISQ values cannot automatically be interpreted as evidence of superior implant survival, prosthetic success, or reduced complication rates. The present cohort was not specifically designed to determine whether longitudinal changes in ISQ are independently associated with improved patient-centered outcomes. RFA measurements should be interpreted within the broader clinical assessment of implant performance. Another consideration relates to the categorization of continuous ISQ values into predefined stability classes. Although the selected thresholds were derived from the established Osstell classification system and are commonly used in clinical practice, threshold-based categorization may result in loss of information and may influence the apparent magnitude of stability-class transitions. For this reason, the primary statistical inferences of the present study were based on continuous ISQ measurements analyzed using mixed-effects regression models. Finally, implant stability was assessed using resonance frequency analysis, which provides an indirect measure of implant‒bone complex stiffness rather than direct evidence of osseointegration. Future prospective studies with longer follow-up should determine whether specific longitudinal ISQ trajectories are associated with clinically relevant patient-centered outcomes.

## Conclusion

 Implant stability increased consistently during healing, reflecting the expected development of secondary biological stability. Anatomical location and implant diameter were the principal determinants of ISQ values, whereas prosthetic configuration and collar design were not independently associated with implant stability.

 Marginal bone remodeling remained limited, survival and success rates were high, and peri-implant soft tissue conditions were generally favorable across all collar designs.

 Longitudinal RFA monitoring appears useful for evaluating implant stability during healing; however, changes in ISQ values were not associated with marginal bone remodeling at the one-year follow-up.

 Prospective studies with longer follow-up are required to determine the clinical significance of different longitudinal ISQ trajectories.

## Competing Interests

 The authors Giovanni Battista Menchini Fabris, Toti Paolo, Trasarti Stefano, Soheil Bechara, and Saverio Cosola declare that they have no known competing financial interests or personal relationships that could have influenced the work reported in this paper. Tommaso Grandi declares that he serves as a consultant for J Dental Care Srl (Modena, Italy).

## Data Availability

 The datasets generated and/or analyzed during the current study are available from the corresponding author on reasonable request.

## Ethical Approval

 The study was approved by the Regional Ethical Review Board of Saint Camillus International University of Health and Medical Sciences (Reference ID: E00930-2023).

## Supplementary Files

Supplementary file 1: Distribution of implants in the 65 patients. Implants with anodized 1.5-mm collar (A), treated collar (T), and other collars (O). Implant type: Icon/Icon Plus (JDI), Icon T (JDT), Pterigo (JDP), and Nasal (JDN).

Supplementary file 2: Patient-level frequencies and percentages of gingival index (GI), modified plaque index (mPI), and modified bleeding index (mBI) scores (0‒3) recorded 1 year after definitive prosthesis delivery. Data were aggregated at the patient level; when multiple implants of the same collar type were present, the worst score (i.e., the maximum value) was retained. Scores were dichotomized (0 vs. ≥ 1) for inferential analysis. Overall comparisons among the three groups were performed using chi-square tests of independence (3 x 2: *) with Bonferroni-adjusted significance thresholds. Prespecified pooled comparing (groups A + B versus group C) were performed using two-side Fisher’s exact test (2 x 2: ^) without multiplicity adjustment.

Supplementary File 3. Figure S1: Orthopantomographic images (patients 1 to 65).
