# Supplementary material for: Longitudinal changes in implant stability assessed by resonance frequency analysis and marginal bone remodeling around immediate implants: A retrospective cohort study
Source: J Adv Periodontol Implant Dent. 2026 Sep 12;18(4):192–204. doi: 10.34172/japid.4325 (PMC13733226; doi:10.34172/japid.4325)
Supplement: Supplementary file 1 [file japid-18-192-s001.pdf]

Menchini-Fabris et al, J Adv Periodontol Implant Dent 2026;18(4):192-S1
doi:10.34172/japid.4325
https://japid.tbzmed.ac.ir

Table Supplementary 1. Distribution of JD implants in the 65 patients. Implants with anodized 1.5-mm collar (A), treated collar (T), and other collars (O). Implant type: Icon/Icon Plus (JDI), Icon T (JDT), Pterigo (JDP), and Nasal (JDN).

pts	age	gender	arch	VIII	VII	VI	V	IV	III	II	I	I	II	III	IV	V	VI	VII	VIII
1	79.6	M	U	-	-	-	-	-	-	-	-	-	-	-	-	-	-	-	-
			L	-	-	T^{JDT} 13x4.5	-	T^{JDT} 13x4.0	-	T^{JDI} 13x3.7	-	-	T^{JDI} 13x3.7	-	T^{JDT} 13x4.0	-	T^{JDT} 13x4.5	-	-
2	69.3	M	U	-	-	T^{JDT} 11.5x4.5	-	T^{JDI} 13x3.7	-	T^{JDI} 13x3.7	-	-	T^{JDI} 13x3.7	-	T^{JDI} 13x3.7	-	T^{JDT} 11.5x4.5	-	-
			L	-	-	T^{JDI} 10x4.3	-	T^{JDI} 10x3.7	-	T^{JDI} 13x3.7	-	-	T^{JDI} 13x3.7	-	T^{JDI} 10x3.7	-	T^{JDI} 8x4.3	-	-
3	64.8	M	U	-	T^{JDI} 13x5.0	-	T^{JDI} 13x4.3	T^{JDI} 15x3.7	-	T^{JDI} 15x3.7	-	-	T^{JDI} 15x3.7	-	-	T^{JDT} 11.5x4.5	-	T^{JDT} 13x4.5	-
			L	-	-	T^{JDT} 10x4.0	-	T^{JDT} 13x4.0	-	-	-	-	T^{JDI} 15x3.7	-	T^{JDI} 13x4.3	-	T^{JDT} 10x4.0	-	-
4	81.1	M	U	-	-	-	-	-	T^{JDI} 15x3.7	-	-	-	-	-	-	-	-	T^{JDI} 13x4.5	-
			L	-	-	-	-	-	-	-	-	-	-	-	-	-	T^{JDI} 13x4.0	-	-
5	60.3	F	U	-	-	T^{JDT} 11.5x4.0	-	T^{JDI} 13x3.7	-	T^{JDI} 18x3.7	-	T^{JDI} 18x3.7	-	T^{JDI} 15x3.7	T^{JDT} 13x4.0	-	-	T^{JDT} 10x4.0	-
			L	-	-	-	T^{JDT} 11.5x4.0	T^{JDT} 13x4.0	-	-	-	-	-	-	-	-	-	-	-
6	71.7	M	U	-	-	-	O^{JDN} 26x4.0	-	-	A^{JDI} 15x3.7	-	-	A^{JDI} 15x3.7	-	-	O^{JDN} 26x4.0	-	-	-
			L	-	-	-	A^{JDI} 15x3.7	-	-	A^{JDI} 15x3.7	-	-	A^{JDI} 15x3.7	-	-	A^{JDI} 15x3.7	-	-	-
7	78.4	M	U	-	T^{JDI} 11.5x4.0	-	T^{JDI} 13x3.7	T^{JDT} 13x3.7	-	-	-	-	-	-	-	-	-	-	-
			L	-	T^{JDT} 13x4.5	-	T^{JDT} 13x4.0	-	-	-	-	-	-	-	-	-	-	-	-
8	44.8	F	U	-	-	T^{JDT} 10x4.5	O^{JDN} 20x4.0	-	-	T^{JDI} 10x3.7	-	-	T^{JDI} 10x3.7	-	T^{JDI} 18x3.7	-	T^{JDT} 10x4.5	-	-
			L	-	A^{JDI} 8x4.3	-	-	A^{JDI} 15x3.7	-	T^{JDI} 10x3.7	-	-	T^{JDI} 10x3.7	-	-	A^{JDI} 15x3.7	-	-	-
9	43.6	M	U	-	-	-	-	-	-	-	-	-	-	-	-	-	-	-	-
			L	-	-	T^{JDI} 11.5x4.3	-	-	-	-	-	-	-	-	-	-	T^{JDI} 11.5x4.3	-	-
10	81.4	M	U	-	-	-	T^{JDT} 10x4.0	-	-	-	-	-	-	-	-	-	T^{JDT} 10x4.0	-	-
			L	-	-	T^{JDT} 10x4.0	-	T^{JDI} 13x3.7	-	-	-	-	-	-	T^{JDI} 13x3.7	-	T^{JDT} 10x4.0	-	-
11	60.2	F	U	-	-	T^{JDT} 11.5x4.5	-	T^{JDT} 13x4.0	-	T^{JDI} 15x3.7	-	-	-	T^{JDI} 15x3.7	-	T^{JDT} 13x4.0	-	T^{JDT} 11.5x4.5	-
			L	-	-	-	-	-	-	-	-	-	-	-	-	-	-	-	-
12	51.0	M	U	-	-	T^{JDT} 11.5x4.0	-	T^{JDT} 13x4.0	-	T^{JDT} 13x4.0	-	-	T^{JDT} 13x4.0	-	T^{JDT} 13x4.0	-	T^{JDT} 11.5x4.0	-	-
			L	-	-	T^{JDT} 11.5x4.0	-	T^{JDT} 13x4.0	-	T^{JDI} 13x3.7	-	-	T^{JDI} 13x3.7	-	T^{JDT} 11.5x4.0	-	T^{JDT} 11.5x4.0	-	-
13	49.2	M	U	-	-	-	T^{JDT} 10x4.5	T^{JDI} 13x3.7	-	T^{JDI} 18x3.7	-	T^{JDI} 18x3.7	-	-	T^{JDI} 15x3.7	-	T^{JDT} 10x4.0	-	-
			L	-	T^{JDT} 11.5x4.0	-	T^{JDT} 11.5x4.0	-	T^{JDI} 18x3.7	-	-	-	-	T^{JDI} 15x3.7	-	T^{JDI} 11.5x3.7	-	T^{JDT} 10x4.0	-
14	72.1	F	U	-	-	-	-	-	-	-	-	-	-	-	-	-	-	-	-
			L	-	-	A^{JDI} 18x4.3	-	-	T^{JDI} 15x3.7	-	-	-	-	-	T^{JDI} 15x3.7	-	A^{JDI} 10x4.3	-	-
15	64.5	M	U	-	-	T^{JDI} 11.5x4.3	-	T^{JDI} 15x3.7	-	T^{JDI} 13x3.7	-	-	T^{JDI} 13x3.7	-	T^{JDI} 13x3.7	-	T^{JDI} 11.5x4.3	-	-
			L	-	-	T^{JDI} 11.5x4.3	-	T^{JDI} 11.5x3.7	-	T^{JDI} 13x3.7	-	-	T^{JDI} 13x3.7	-	T^{JDI} 11.5x3.7	-	T^{JDI} 11.5x4.3	-	-
16	45.7	M	U	-	-	T^{JDI} 10x4.3	-	T^{JDI} 11.5x3.7	-	-	T^{JDI} 15x3.7	-	-	-	T^{JDI} 11.5x4.3	-	T^{JDI} 8x5.0	-	-
			L	-	-	-	-	-	-	T^{JDI} 13x3.7	-	-	-	-	-	-	-	-	-
17	45.0	M	U	-	-	T^{JDI} 10x4.3	-	T^{JDI} 13x3.7	-	T^{JDI} 15x3.7	-	-	T^{JDI} 18x3.7	-	T^{JDI} 15x3.7	-	T^{JDI} 10x4.3	-	-
			L	-	-	-	-	-	-	-	-	-	-	-	-	-	-	-	-
18	59.7	F	U	-	-	-	T^{JDI} 8x5.0	-	T^{JDI} 11.5x3.7	T^{JDI} 18x3.7	-	-	-	-	-	-	-	-	-
			L	-	A^{JDI} 10x4.3	-	T^{JDI} 10x3.7	-	-	-	-	-	-	-	T^{JDI} 10x4.3	-	T^{JDI} 10x3.7	-	-
19	33.1	M	U	-	-	-	-	-	-	-	-	-	T^{JDI} 18x3.7	-	-	-	-	-	-
			L	-	-	-	T^{JDI} 11.5x3.7	-	-	-	-	-	-	-	-	-	T^{JDI} 11.5x4.3	T^{JDI} 11.5x4.3	-
20	43.0	F	U	-	-	T^{JDI} 10x3.7	-	T^{JDI} 11.5x3.7	-	-	-	-	-	-	-	T^{JDI} 11.5x3.7	T^{JDI} 10x4.3	-	-
			L	-	-	T^{JDI} 11.5x4.3	-	T^{JDI} 11.5x3.7	-	-	-	-	-	-	-	-	-	-	-
21	51.5	F	U	-	-	T^{JDT} 10x4.5	-	T^{JDT} 10x4.0	T^{JDI} 13x3.7	-	-	-	-	-	T^{JDI} 13x3.7	-	T^{JDT} 10x4.5	-	-
			L	-	-	-	-	-	-	-	-	-	-	-	-	-	-	-	-
22	44.5	M	U	-	-	T^{JDT} 11.5x4.0	-	-	T^{JDI} 15x3.7	-	T^{JDI} 15x3.7	T^{JDI} 15x3.7	-	T^{JDI} 15x3.7	-	T^{JDT} 10x4.5	T^{JDT} 10x4.0	-	-
			L	-	-	A^{JDI} 11.5x4.3	-	T^{JDI} 11.5x3.7	-	T^{JDI} 13x3.7	-	-	T^{JDI} 13x3.7	-	T^{JDT} 11.5x4.0	-	T^{JDT} 11.5x4.0	-	-
23	51.9	F	U	-	-	T^{JDT} 10x4.0	T^{JDT} 10x4.5	-	-	T^{JDI} 15x3.7	-	-	T^{JDI} 15x3.7	-	T^{JDI} 10x3.7	-	T^{JDT} 11.5x4.5	-	-
			L	-	-	-	-	-	-	-	-	-	-	-	T^{JDT} 13x4.0	-	T^{JDT} 10x4.0	T^{JDT} 10x4.5	-
24	39.9	F	U	-	T^{JDT} 13x4.5	-	T^{JDT} 13x4.5	-	-	T^{JDI} 15x3.7	-	-	-	-	T^{JDT} 13x4.0	T^{JDT} 13x4.0	T^{JDT} 11.5x4.5	T^{JDT} 11.5x4.5	-
			L	-	T^{JDI} 10x4.3	T^{JDI} 10x4.3	-	-	-	-	-	-	-	-	-	-	T^{JDT} 10x4.0	T^{JDT} 10x4.0	-
25	44.8	M	U	-	-	-	-	-	-	-	-	-	-	-	-	-	T^{JDI} 13x3.7	-	-
			L	-	T^{JDI} 11.5x5.0	T^{JDI} 11.5x4.3	-	-	-	-	-	-	-	-	-	-	T^{JDT} 13x4.0	-	-
26	69.5	F	U	-	-	-	-	-	-	-	-	-	-	-	-	-	-	-	-
			L	-	-	T^{JDT} 10x4.5	-	T^{JDT} 10x4.0	-	-	-	-	-	T^{JDI} 13x3.7	T^{JDI} 13x3.7	-	T^{JDI} 10x5.0	-	-
27	25.1	M	U	-	-	-	-	-	-	-	-	-	T^{JDI} 15x3.7	-	-	-	T^{JDT} 13x4.5	T^{JDT} 11.5x4.0	-
			L	-	-	-	T^{JDI} 11.5x3.7	-	-	-	-	-	-	-	-	T^{JDT} 13x4.0	T^{JDI} 13x4.3	-	-
28	53.4	M	U	-	-	-	-	-	-	-	-	-	-	-	T^{JDT} 10x4.0	T^{JDT} 10x4.0	-	T^{JDT} 8x4.0	-
			L	-	-	-	-	-	-	-	-	-	-	-	T^{JDI} 10x3.7	-	-	T^{JDI} 10x3.7	-
29	56.7	M	U	-	T^{JDT} 10x4.0	-	-	-	-	-	-	-	-	-	-	-	-	-	-
			L	-	-	T^{JDI} 13x4.3	-	-	-	-	-	-	-	-	-	T^{JDT} 13x4.0	T^{JDT} 13x4.0	-	-
30	42.7	M	U	-	-	T^{JDT} 10x4.5	-	T^{JDI} 15x3.7	-	T^{JDI} 15x3.7	-	-	T^{JDI} 15x3.7	-	T^{JDI} 15x3.7	-	T^{JDT} 10x4.5	-	-
			L	-	-	T^{JDT} 11.5x4.0	-	T^{JDI} 15x3.7	-	T^{JDI} 15x3.7	-	-	-	T^{JDT} 15x4.3	T^{JDT} 13x4.0	-	T^{JDT} 13x4.0	-	-
31	76.1	F	U	-	-	T^{JDT} 11.5x4.5	-	O^{JDN} 22x4.0	-	T^{JDI} 15x3.7	-	A^{JDI} 18x3.7	-	-	A^{JDI} 18x3.7	-	T^{JDT} 10x4.0	-	-
			L	-	T^{JDI} 8x3.7	-	A^{JDI} 18x3.7	-	-	O^{JDN} 20x4.0	-	-	O^{JDN} 20x4.0	-	-	A^{JDI} 18x3.7	-	T^{JDI} 8x3.7	-

32	39.7	F	U	-	-	-	-	-	-	-	-	-	-	-	-	T^{JDT} 11.5x4.0	T^{JDT} 11.5x4.5	-	T^{JDT} 11.5x4.0
			L	-	-	-	-	-	-	-	-	-	-	-	-	-	-	-	-
33	33.1	M	U	-	-	-	-	-	-	-	-	-	-	-	-	T^{JDT} 11.5x4.5	-	-	-
			L	-	-	-	-	-	-	-	-	-	-	-	-	-	-	T^{JDI} 11.5x4.3	-
34	42.3	F	U	-	-	T^{JDT} 13x4.0	-	-	-	-	-	-	-	-	-	-	-	-	-
			L	-	-	T^{JDI} 10x3.7	-	-	-	-	-	-	-	-	-	-	-	-	-
35	46.6	F	U	-	-	-	T^{JDT} 13x4.5	T^{JDI} 15x4.3	-	T^{JDI} 15x3.7	-	-	T^{JDI} 15x3.7	-	-	T^{JDI} 13x3.7	T^{JDI} 11.5x4.3	-	-
			L	-	-	-	-	-	-	-	-	-	-	-	-	-	-	-	-
36	57.8	M	U	-	-	T^{JDT} 10x4.0	-	T^{JDI} 13x3.7	-	-	-	-	-	-	T^{JDT} 11.5x4.0	-	T^{JDT} 10x4.0	-	-
			L	-	-	T^{JDI} 13x4.3	-	-	-	-	-	-	-	-	-	-	T^{JDI} 11.5x4.3	-	-
37	66.2	M	U	-	T^{JDT} 10x4.0	-	T^{JDT} 13x3.7	T^{JDI} 13x4.0	-	-	-	-	-	-	-	-	T^{JDT} 11.5x4.0	-	-
			L	-	-	T^{JDT} 10x4.0	-	-	-	-	-	-	-	-	-	-	-	-	-
38	57.5	F	U	-	-	T^{JDT} 10x4.0	-	T^{JDT} 11.5x4.0	-	-	-	-	-	-	-	-	T^{JDT} 10x4.0	-	-
			L	-	-	T^{JDT} 10x4.0	-	-	-	-	-	-	-	-	-	-	T^{JDT} 10x4.5	-	-
39	59.2	F	U	-	-	-	-	-	-	-	-	-	-	-	T^{JDT} 13x4.0	-	T^{JDT} 10x4.0	-	-
			L	-	-	-	-	-	-	-	-	-	-	-	-	-	-	-	-
40	46.6	F	U	-	T^{JDT} 10x4.5	-	-	T^{JDI} 18x3.7	-	-	-	-	-	-	-	-	-	-	-
			L	-	-	-	-	-	-	-	-	-	-	-	-	-	-	-	-
41	45.4	F	U	-	-	-	-	-	-	-	-	-	-	-	-	-	A^{JDI} 8x4.3	-	-
			L	-	-	T^{JDI} 10x4.3	-	T^{JDI} 13x4.3	-	A^{JDI} 13x3.7	-	-	T^{JDI} 13x3.7	-	T^{JDI} 13x4.3	-	A^{JDI} 10x4.3	-	-
42	62.0	M	U	-	-	-	-	-	-	-	-	-	-	-	-	-	-	-	-
			L	-	-	-	-	-	-	T^{JDI} 10x3.7	-	-	T^{JDI} 10x3.7	T^{JDI} 10x3.7	-	-	-	-	-
43	64.5	F	U	O^{JDP} 18x3.3	-	T^{JDI} 13x3.7	-	A^{JDI} 13x3.7	-	-	-	-	-	-	A^{JDI} 11.5x3.7	A^{JDI} 11.5x3.7	-	-	O^{JDP} 18x3.3
			L	-	-	A^{JDI} 11.5x3.7	-	-	-	-	T^{JDI} 13x3.7	-	-	-	-	-	A^{JDI} 11.5x3.7	-	-
44	61.6	F	U	-	-	-	-	-	-	-	-	-	-	-	-	-	-	-	-
			L	-	-	-	-	-	A^{JDI} 11.5x3.7	-	T^{JDI} 13x3.2	T^{JDI} 13x3.2	-	T^{JDI} 11.5x3.7	-	-	-	-	-
45	62.1	F	U	-	-	-	-	-	-	-	-	-	-	-	T^{JDI} 10x3.7	T^{JDI} 10x4.3	-	O^{JDP} 15x3.3	-
			L	-	-	-	-	-	-	-	-	-	-	-	-	-	-	-	-
46	71.7	M	U	O^{JDP} 15x3.3	-	-	T^{JDI} 10x3.7	-	-	-	A^{JDI} 11.5x3.7	T^{JDI} 11.5x3.7	-	-	A^{JDI} 8x3.7	-	-	-	O^{JDP} 15x3.3
			L	-	-	-	-	-	-	-	-	-	-	-	-	-	-	-	-
47	62.9	M	U	-	T^{JDI} 10x4.3	-	A^{JDI} 11.5x3.7	-	-	-	A^{JDI} 11.5x3.7	T^{JDI} 11.5x3.7	A^{JDI} 11.5x3.7	-	-	T^{JDI} 11.5x3.7	-	T^{JDI} 10x4.3	-
			L	-	-	-	-	-	-	-	-	-	-	-	-	-	-	-	-
48	85.5	M	U	-	-	-	-	-	-	-	-	-	-	-	-	-	-	-	-
			L	-	-	A^{JDI} 8x3.7	-	A^{JDI} 11.5x3.7	-	A^{JDI} 13x3.7	-	-	A^{JDI} 13x3.7	-	T^{JDI} 11.5x3.7	-	T^{JDI} 10x3.7	-	-
49	66.2	F	U	-	T^{JDI} 13x4.3	-	-	A^{JDI} 13x3.7	A^{JDI} 13x3.3	-	T^{JDI} 13x3.3	T^{JDI} 13x3.7	-	-	-	-	-	-	-
			L	-	T^{JDI} 10x4.3	-	-	A^{JDI} 11.5x3.7	-	-	-	-	-	-	A^{JDI} 10x3.7	-	-	T^{JDI} 10x4.3	-
50	68.5	M	U	-	-	A^{JDI} 8x3.7	T^{JDI} 11.5x3.7	A^{JDI} 13x3.7	-	-	-	-	-	-	-	-	-	-	-
			L	-	-	A^{JDI} 10x4.3	-	-	-	-	-	-	-	-	-	-	T^{JDI} 10x4.3	-	-
51	56.8	F	U	-	A^{JDI} 10x3.7	-	-	T^{JDI} 10x4.3	-	-	-	-	-	-	-	T^{JDI} 13x3.7	-	-	A^{JDI} 10x3.7
			L	-	-	-	-	-	-	-	-	-	-	-	-	-	-	-	-
52	75.6	M	U	-	-	-	T^{JDI} 13x3.7	-	T^{JDI} 13x3.7	-	T^{JDI} 13x3.7	-	T^{JDI} 13x3.7	T^{JDI} 13x3.7	-	T^{JDI} 13x3.7	-	-	O^{JDP} 18x3.3
			L	-	-	T^{JDI} 10x4.3	-	T^{JDI} 11.5x4.3	-	T^{JDI} 11.5x4.3	-	-	T^{JDI} 11.5x4.3	-	T^{JDI} 11.5x4.3	-	T^{JDI} 10x4.3	-	-
53	60.9	F	U	-	-	-	-	-	-	-	-	-	-	-	-	-	-	-	-
			L	-	-	-	A^{JDI} 11.5x3.7	-	-	A^{JDI} 13x3.7	-	T^{JDI} 13x3.7	-	-	T^{JDI} 11.5x4.3	-	-	-	-
54	44.1	M	U	-	-	-	T^{JDI} 11.5x3.7	-	T^{JDI} 11.5x3.7	-	T^{JDI} 11.5x3.7	A^{JDI} 10x3.7	-	T^{JDI} 11.5x3.7	-	-	T^{JDI} 11.5x3.7	-	-
			L	-	-	T^{JDI} 10x3.7	-	-	-	-	T^{JDI} 13x3.7	-	T^{JDI} 13x3.7	-	-	T^{JDI} 10x3.7	-	-	-
55	49.7	M	U	-	T^{JDI} 11.5x4.3	-	A^{JDI} 8x4.3	T^{JDI} 11.5x3.7	-	A^{JDI} 13x3.7	T^{JDI} 13x3.7	-	T^{JDI} 11.5x3.7	-	A^{JDI} 11.5x3.7	-	-	-	-
			L	-	-	-	-	-	-	-	-	-	-	-	-	-	-	-	-
56	62.5	F	U	-	-	-	-	-	-	-	-	-	-	-	-	-	-	-	-
			L	-	-	-	-	-	A^{JDI} 13x3.7	-	A^{JDI} 13x3.7	A^{JDI} 13x3.7	-	A^{JDI} 13x3.7	-	-	-	-	-
57	59.7	F	U	-	-	-	-	-	-	-	-	-	-	-	-	-	-	-	-
			L	-	-	-	-	-	T^{JDI} 15x3.7	-	A^{JDI} 13x3.7	A^{JDI} 13x3.7	-	T^{JDI} 15x3.7	-	-	-	-	-
58	62.5	M	U	-	-	-	-	-	-	-	-	-	-	-	-	-	-	-	-
			L	-	-	T^{JDI} 10x3.7	-	-	T^{JDI} 15x3.7	-	A^{JDI} 15x3.7	T^{JDI} 15x3.7	-	A^{JDI} 15x3.7	-	-	T^{JDI} 10x3.7	-	-
59	71.0	F	U	-	-	-	-	-	-	-	-	-	-	-	-	-	-	-	-
			L	-	-	-	-	A^{JDI} 13x3.7	-	T^{JDI} 13x3.7	-	-	T^{JDI} 13x3.7	-	A^{JDI} 13x3.7	-	-	-	-
60	75.0	M	U	-	-	T^{JDI} 11.5x4.3	-	-	T^{JDI} 11.5x4.3	-	A^{JDI} 13x3.7	T^{JDI} 13x3.7	-	T^{JDI} 11.5x4.3	-	-	T^{JDI} 11.5x4.3	-	-
			L	-	-	T^{JDI} 10x4.3	T^{JDI} 10x4.3	-	-	-	-	-	-	-	-	A^{JDI} 10x3.7	A^{JDI} 10x3.7	-	-
61	77.4	M	U	-	-	A^{JDI} 8x4.3	-	-	T^{JDI} 15x3.7	-	-	-	-	-	-	-	T^{JDI} 8x4.3	-	-
			L	-	A^{JDI} 10x3.7	-	-	T^{JDI} 15x3.7	-	T^{JDI} 15x3.7	-	T^{JDI} 15x3.7	-	T^{JDI} 15x3.7	-	-	-	T^{JDI} 10x4.3	-
62	67.8	F	U	O^{JDP} 15x3.3	-	-	-	A^{JDI} 13x3.7	-	-	-	-	-	-	-	T^{JDI} 13x4.3	-	-	O^{JDP} 15x3.3
			L	-	T^{JDI} 8x3.7	T^{JDI} 8x3.7	-	-	-	-	-	-	-	-	-	-	-	-	-
63	53.5	F	U	-	T^{JDI} 11.5x3.7	-	A^{JDI} 13x3.7	-	-	A^{JDI} 13x3.7	-	-	T^{JDI} 11.5x3.7	-	-	A^{JDI} 13x3.7	-	A^{JDI} 11.5x3.7	-
			L	-	-	-	-	-	-	-	-	-	-	-	-	-	-	-	-
64	57.0	M	U	-	O^{JDP} 18x3.3	-	T^{JDI} 8x4.3	-	-	-	-	-	-	-	-	T^{JDI} 8x4.3	-	O^{JDP} 18x3.3	-
			L	-	-	T^{JDI} 6x4.3	T^{JDI} 6x4.3	-	-	-	-	-	-	-	-	-	A^{JDI} 6x4.3	-	-
65	81.0	F	U	-	-	T^{JDI} 10x3.7	-	A^{JDI} 13x3.7	-	T^{JDI} 11.5x4.3	-	-	T^{JDI} 11.5x4.3	-	T^{JDI} 13x3.7	-	A^{JDI} 10x3.7	-	-
			L	-	-	-	-	-	T^{JDI} 10x3.7	-	T^{JDI} 10x3.7	T^{JDI} 10x3.7	-	T^{JDI} 10x3.7	-	-	-	-	-
