# Supplementary material for: Longitudinal changes in implant stability assessed by resonance frequency analysis and marginal bone remodeling around immediate implants: A retrospective cohort study
Source: J Adv Periodontol Implant Dent. 2026 Sep 12;18(4):192–204. doi: 10.34172/japid.4325 (PMC13733226; doi:10.34172/japid.4325)
Supplement: Supplementary file 2 [file japid-18-192-s002.pdf]

Menchini-Fabris et al, J Adv Periodontol Implant Dent 2026;18(4):192-S2
doi:10.34172/japid.4325
https://japid.tbzmed.ac.ir

Table Supplementary 2. Patient-level frequencies and percentages of gingival index (GI), modified plaque index (mPI), and modified bleeding index (mBI) scores (0-3) recorded 1 years after definitive prosthesis delivery. Data were aggregated at the patient level; when multiple implants of the same collar type were present, the worst score (maximum value) was retained. Scores were dichotomized (0 vs. ≥1) for inferential analysis. Overall comparisons among the three groups were performed using chi-square tests of independence (3 x 2: *) with Bonferroni-adjusted significance thresholds. Prespecified pooled comparing (Groups A + B versus Group C) were performed using two-side Fisher's exact test (2 x 2: ^) without multiplicity adjustment.

variable	implant groups	n		1 year score			
				**0**	**1**	**2**	**3**
gingival index	Group A (treated collar type 1)	37	count	15	20	2	0
			percent	40.5	54.1	5.4	0
	Group B (treated collar type 2)	11	count	7	3	1	0
			percent	63.6	27.3	9.1	0
	Group C (anodized collar)	17	count	12	4	1	0
			percent	70.6	23.5	5.9	0
primary analysis	Group A & Group B & Group C	37 & 11 & 17	p-value	0.0864*			
pooled analysis	Group A + Group B vs Group C	48 *vs*. 17	p-value	0.0964^			
modified plaque index	Group A (treated collar type 1)	37	count	12	18	7	0
			percent	32.4	48.6	18.9	0
	Group B (treated collar type 2)	11	count	6	4	1	0
			percent	54.5	36.4	9.1	0
	Group C (anodized collar)	17	count	7	9	1	0
			percent	41.2	52.9	5.9	0
primary analysis	Group A & Group B & Group C	37 & 11 & 17	p-value	0.4018*			
pooled analysis	Group A + Group B vs Group C	48 *vs*. 17	p-value	0.7808^			
modified bleeding index	Group A (treated collar type 1)	37	count	19	9	9	0
			percent	51.4	24.3	24.3	0
	Group B (treated collar type 2)	11	count	5	4	2	0
			percent	45.5	36.4	18.2	0
	Group C (anodized collar)	17	count	14	3	0	0
			percent	82.4	17.6	0	0
primary analysis	Group A & Group B & Group C	37 & 11 & 17	p-value	0.0629*			
pooled analysis	Group A + Group B vs Group C	48 *vs*. 17	p-value	**0.0239**^			
