# Supplementary material for: Longitudinal changes in implant stability assessed by resonance frequency analysis and marginal bone remodeling around immediate implants: A retrospective cohort study
Source: J Adv Periodontol Implant Dent. 2026 Sep 12;18(4):192–204. doi: 10.34172/japid.4325 (PMC13733226; doi:10.34172/japid.4325)
Supplement: Supplementary file 3 [file japid-18-192-s003.pdf]

Menchini-Fabris et al, J Adv Periodontol Implant Dent 2026;18(4):192-S3
doi:10.34172/japid.4325
https://japid.tbzmed.ac.ir

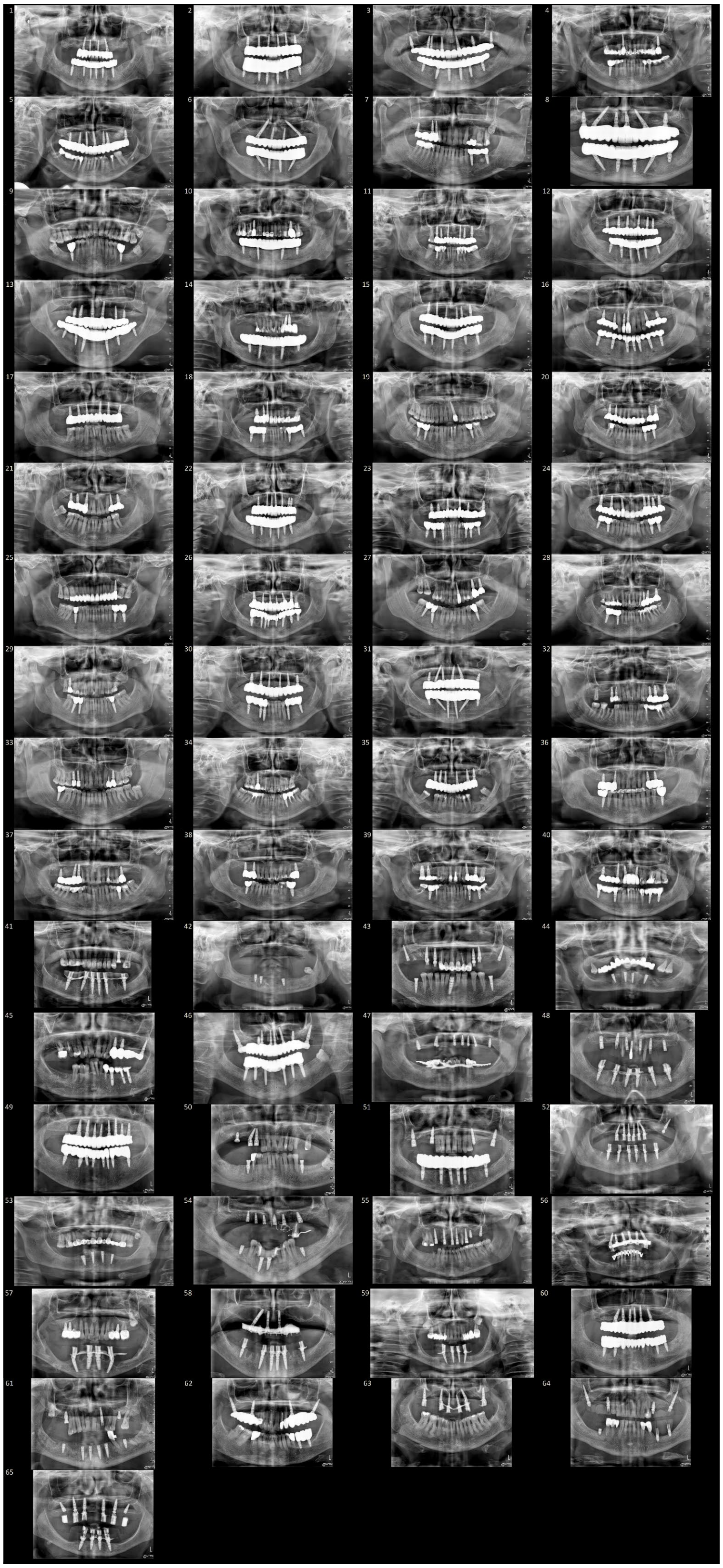

Figure S1: Orthopantomographic images (patients 1 to 65).
